# Weight suppression in bulimia nervosa: relationship with cognitive behavioral therapy outcome

**DOI:** 10.1186/2050-2974-1-S1-O10

**Published:** 2013-11-14

**Authors:** Hayley Dawkins, Hunna Watson, Sarah Egan, Robert Kane, Stephanie Hill

**Affiliations:** 1 School of Psychology and Speech Pathology, Curtin University; 2 Centre for Clinical Interventions, Department of Health in Western Australia

## 

Weight suppression has been found to negatively predict treatment completion of cognitive behavioural therapy (CBT). However, subsequent attempts to replicate these findings have failed. In light of this, the current study revisits the relationship between weight suppression and treatment outcomes in bulimia nervosa. We propose that moderator effects may assist in interpreting previous inconsistency. Moderators tested were parent history of overweight, chubby/overweight childhood body shape, higher pre-treatment body mass index, and highest ever adult weight discrepancy. Participants were 117 female outpatients aged 16-54 years with bulimic disorders treated with enhanced cognitive behavioural therapy at a specialist community clinic. Logistic regression indicated that pre-treatment weight suppression did not predict drop-out or poor treatment outcome. No moderator effects were observed when hypothesised moderator features were included in treatment completion or treatment outcome models. The current study calls into question the association between weight suppression and treatment outcome. Future research into moderator models, using a larger sample, could assist us to refine conceptualizations of why some patients who have a weight suppression history are vulnerable to poor treatment adherence and outcome and to establish clinical interventions that enhance prognosis.

This abstract was presented in the **Adult Treatment and Services** stream of the 2013 ANZAED Conference.

